# Visual Functioning and Progression from Intermediate Age-Related Macular Degeneration (AMD) to Advanced AMD

**DOI:** 10.1080/09286586.2026.2702462

**Published:** 2026-07-23

**Authors:** Shruti Patel, Talisa E. de Carlo Forest, Andres Lisker-Cervantes, Ramya Gnanaraj, Anne M. Lynch, Naresh Mandava, Marc T. Mathias, Niranjan Manoharan, Jennifer L. Patnaik

**Affiliations:** Department of Ophthalmology, University of Colorado School of Medicine, Aurora, Colorado, USA

**Keywords:** Age-related macular degeneration (AMD), NEI VFQ-25, neovascular AMD (NV AMD), non-neovascular AMD (NNV AMD), progression, visual functioning

## Abstract

**Purpose:**

To investigate whether the National Eye Institute Visual Function Questionnaire-25 (NEI VFQ-25) can predict the progression of intermediate age-related macular degeneration (iAMD) to advanced non-neovascular (NNV) or neovascular (NV).

**Methods:**

380 patients aged 55–99 years with iAMD in one or both eyes and no evidence of geographic atrophy (GA) or NV AMD in either eye were enrolled (2014–2023 into the University of Colorado AMD registry) with follow-up through November 2024. AMD classification and progression were based on multimodal imaging review by two vitreoretinal surgeons. Baseline visual acuity (LogMAR) and VFQ-25 scores were collected at enrollment. Cox proportional hazards models assessed time to progression. Kaplan-Meier analysis evaluated significant VFQ-25 subscales.

**Results:**

Among 380 iAMD participants (mean age 76.1 years, 95.8% white), 131 (34.5%) progressed to advanced AMD over a median 32-month follow up (SD- 27). Worse baseline LogMAR in both the better(HR: 6.39, 95% CI: 1.57–26; *p* = 0.001) and worse- (HR: 2.64, 95% CI: 1.26–5.55; *p* = 0.010) seeing eyes predicted progression. Lower baseline VFQ-25 general vision (HR: 0.84 per 10-point increase, 95% CI: 0.74–0.95, *p* = 0.006) and distance activities scores (HR: 0.89 per 10-point increase, 95% CI: 0.80–0.98, *p* = 0.019) predicted progression during follow-up. In multivariable analysis adjusting for age and LogMAR for both eyes, this remained significant for general vision (HR: 0.85, 95% CI:0.75–0.98, *p* = 0.022). Kaplan-Meier analysis showed faster progression for patients with baseline general vision and distance activity subscores below 85 (*p* = 0.013 and *p* = 0.011, respectively).

**Conclusion:**

Lower NEI-VFQ-25 general vision subscale scores independently predicted progression to advanced AMD.

## Introduction

Age-related macular degeneration (AMD) is the leading cause of irreversible severe vision impairment among older adults in many high-income countries.^1^ Approximately 200 million people worldwide are living with an active AMD diagnosis, which can be classified into two main types: non-neovascular (NNV) and neovascular (NV).^2^

NNV AMD is further subdivided into three stages: early, intermediate, and advanced.^2^ With aging, the macula undergoes increased oxidative stress, which leads to the buildup of cellular debris and lipids between the retinal pigment epithelium (RPE) and Bruch’s membrane.^2^ Ultimately, inflammation results in the death of photoreceptors, RPE, and choriocapillaris, leading to geographic atrophy (GA), which is characteristic of advanced NNV AMD.^3^ In contrast, NV AMD is characterized by the presence of choroidal neovascularization (CNV). Both advanced phenotypes cause severe vision impairment.^4^ Patients with advanced AMD often experience a notable decline in their quality of life, as they lose independence in tasks like driving.

The National Eye Institute Visual Function Questionnaire 25 (NEI VFQ-25) is a tool that uses patient-reported outcomes to assess their visual functioning. The comprehensive questionnaire includes factors such as general vision, difficulty with near vision activities, difficulty with distance vision activities, challenges in social functioning, role limitations, dependency, mental health symptoms, driving difficulties, peripheral vision, color vision, ocular pain, and general health to quantify visual burden on a patient with chronic eye disease.^5^ The VFQ-25 has been established in the literature as a valid and reliable method for tracking patients’ visual impairment. Other studies have explored risk factors that help predict AMD progression, including demographic, environmental, genetic, imaging, and molecular factors.^6^ However, the utility of the VFQ-25 in predicting progression to either advanced NNV AMD or NV AMD remains poorly understood.

Our prior work demonstrated that both the type of advanced AMD and the number of eyes affected visual function as measured by VFQ-25 scores.^7^ We also found that higher VFQ-25 subscale scores for general health and driving were associated with a reduced risk of mortality in a Colorado cohort of AMD patients.^8^ Building on our previous research on the role of the VFQ in AMD, we designed this study to assess whether the composite score or components of the VFQ-25 predict the progression from intermediate AMD (iAMD) to advanced NNV AMD or NV AMD. Given the significant decline in quality of life for patients with advanced AMD, early detection through a standardized screening tool like the VFQ-25 could allow healthcare providers and patients to be more aware of the risk of progression.

## Materials and methods

### AMD registry

The Department of Ophthalmology at the University of Colorado School of Medicine established a patient registry in 2014 for individuals at the Sue Anschutz-Rodgers Eye Center and CU optometry clinics.^9^ The current prospective cohort study is based on patients enrolled in the registry between July 2014 and May 2023, with follow-up through November 2024. The Colorado Multiple Institutional Review Board approved the registry, and the study adheres to the principles of the Declaration of Helsinki. Informed consent was obtained in writing from each participant. The registry collects comprehensive data for each patient, including demographics, clinical characteristics, and imaging records. Additionally, patients complete the NEI VFQ-25 upon enrollment.

### Inclusion and exclusion criteria for the present study

Patients ages 55 to 99 years with iAMD in at least one eye who had the capacity to provide informed consent were included. Exclusion criteria was defined by any of the following: terminal illness, ocular inflammatory disease, prior treatment with anti-vascular endothelial growth factor injections, other macular disease, ocular melanoma, corneal transplant, current systemic cancer treatment, or significant mental health conditions/advanced dementia. Patients with advanced NNV AMD or CNV in either eye at enrollment were excluded.

### Classification of AMD

AMD stage at enrollment and at final follow-up was determined by two vitreo-retinal specialists using the Beckman classification system for Age-Related Macular Degeneration (AMD), via multimodal imaging.^10,11^ Any discrepancy in classification was resolved by a third vitreo-retinal specialist. The imaging modalities included spectral-domain optical coherence tomography (SD-OCT), fundus autofluorescence (FAF), and color fundus photography of the macula.

### Visual acuity

At enrollment, a certified ophthalmic technician evaluated each patient’s habitual visual acuity (HVA) using Snellen charts. The results were then converted to corresponding LogMAR values for statistical analysis. The eye with the lower LogMAR value was designated as the “better-seeing eye,” while the eye with the higher LogMAR value was considered the “worse-seeing eye.”

### NEI VFQ-25

Upon enrollment, each study participant also completed the NEI VFQ-25. The NEI VFQ-25 provides scores for each subscale and a composite score, which is an average of the vision-related subscale scores, excluding general health.^12^ Subscale scores vary from 0 to 100, with 0 indicating lowest visual functioning and 100 demonstrating the highest possible visual functioning.^12^

### Statistical analysis

The primary outcome of progression to advanced AMD included either progression to the advanced form of NNV and/or to NV in at least one eye. Progression rates for sub-categories are presented as basic frequencies and proportions. Means, standard deviations (SD), medians and interquartile ranges (IQR) are presented by progression status for age, LogMAR values for both eyes, the VFQ-25 composite score, and for the 12 subscales. Cox proportional hazards modeling was used to assess time to event for progression to either advanced form with composite score or subscale scores as independent variables in separate models. Progression to advanced NNV AMD and NV AMD were assessed separately with the first type of progression identified for this analysis. The proportionality assumption was tested by including a time by predictor variable in the models. Hazards ratios, 95% confidence intervals, and *p*-values are presented for univariate and multivariable models. Multivariable models included age as a clinically relevant variable and significant variables in the univariate model. Spearman correlation coefficients were used to assess the correlation between variables included in the multivariable model. Kaplan-Meier curves of time to progression are presented for subscale scores that were significant, with categories of scoring 85 or greater or less than 85. The Logrank test was used to compare the categorized VFQ score groups.

## Results

A total of 436 participants were initially enrolled in the study. Of these, 56 participants were excluded due to incomplete VFQ-25 survey data, resulting in 380 participants being included in the final analysis. The average follow-up time for this cohort was 39 (SD: 27) months with a median of 32 months.

Table 1 provides detailed information regarding participant demographics at enrollment. The study population was predominantly white, accounting for 95.8% of participants. Among the 380 patients, the average age was 76.1 (SD: 7.1). Of the 380 participants, 131 (34.5%) progressed to advanced AMD in at least one eye at some point in the study. Among these 131 patients who converted, 69 (52.7%) were to advanced NNV, 44 (33.6%) to advanced NV, and 18 (13.7%) patients converted in one eye to NNV and one eye to NV. When comparing patients who did and did not progress to advanced AMD, older age and worse LogMAR values, for the better-seeing eye were significantly associated with progression (HR: 6.39, 95% CI: 1.57–26; *p* = 0.001). LogMAR values for the worse-seeing eye were also significantly associated with progression (HR: 2.64, 95% CI: 1.26–5.55; *p* = 0.010), although the hazard ratio was lower than for the better-seeing eye.

Table 2 compares VFQ-25 composite and subscale scores between participants who did and did not convert to advanced AMD. In separate univariate models of VFQ-25 subscales, general vision emerged as a significant predictor of progression (*p* = 0.006). Specifically, for every 10-point increase in the general vision subscale score, there was a 16% lower risk of progression (HR: 0.84, 95% CI: 0.74–0.95). Distance activities were also identified as a significant predictor of progression (*p* = 0.019) with an 11% lower risk of progression for every 10-point increase in the distance activities subscale score (HR: 0.89, 95% CI: 0.80–0.98). Furthermore, the composite score approached statistical significance in predicting AMD progression (HR: 0.86, 95% CI: 0.74–1.02; *p* = 0.075). However, the near activities subscale was not significantly (*p* = 0.092) associated with progression (HR: 0.92, 95% CI: 0.83–1.01). Interestingly, the mental health subscale demonstrated a near-significant (*p* = 0.064) association with AMD progression (HR: 0.91, 95% CI: 0.82–1.01). Spearman correlation coefficients were significant (*p* < 0.0001) for the better-seeing and worse-seeing eye for general vision (−0.32 and −0.30, respectively) and for distance activities (−0.26 and −0.21, respectively).

To further evaluate the predictive value of general vision and distance activities, a multivariable analysis was conducted to control for potential confounding factors, including age and LogMAR values, for both the better-seeing and worse-seeing eyes (Table 3). After adjustment, only general vision maintained significance in predicting progression with an adjusted hazard ratio similar to unadjusted (HR: 0.85, 95% CI:0.75–0.98, *p* = 0.022). No other factors, including the subscale of distance activities, remained significant in the multivariable model.

**Figure 1.**
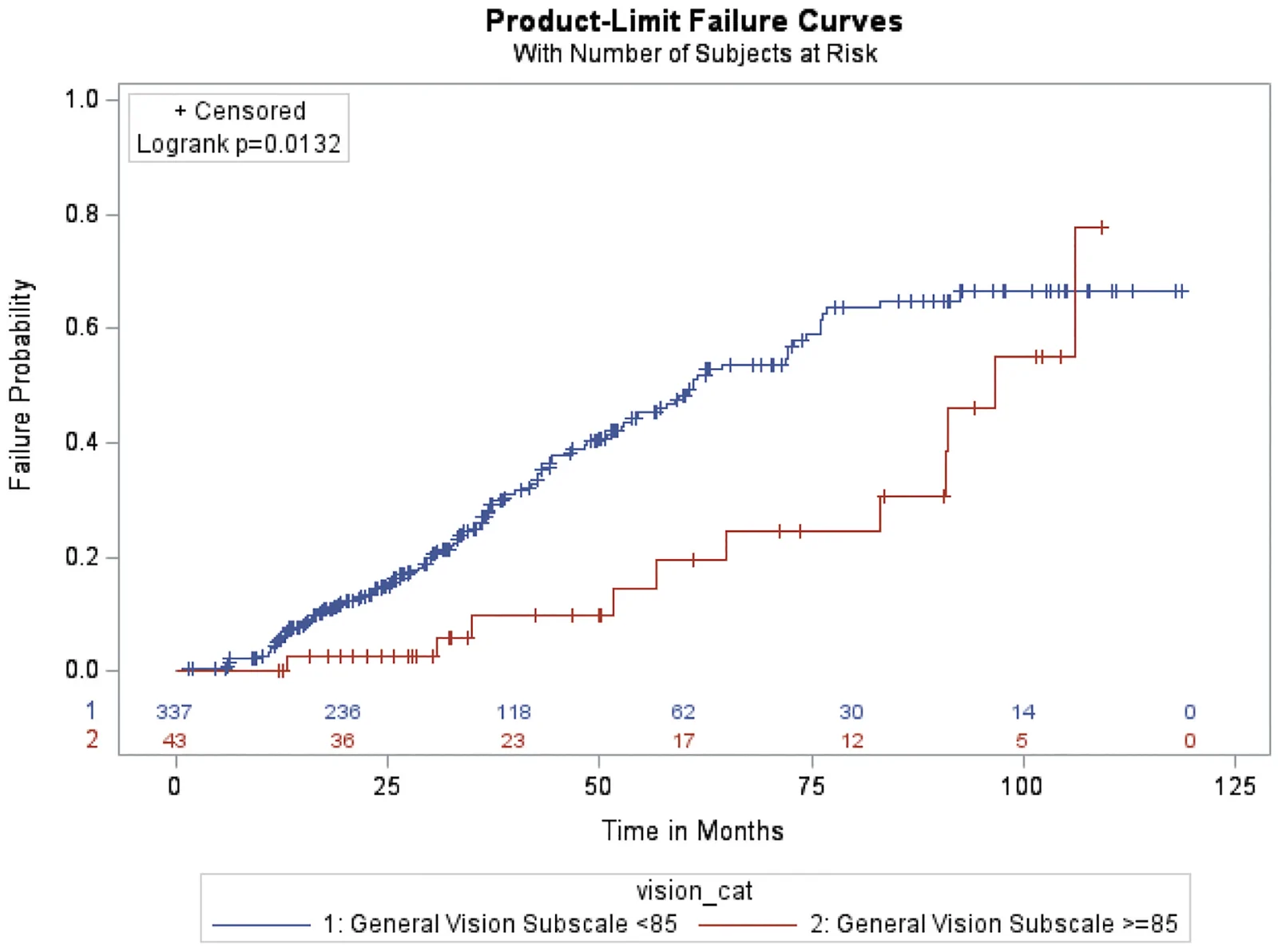
Kaplan-Meier curve of progression to advanced AMD by subscale score of general vision.

**Figure 2.**
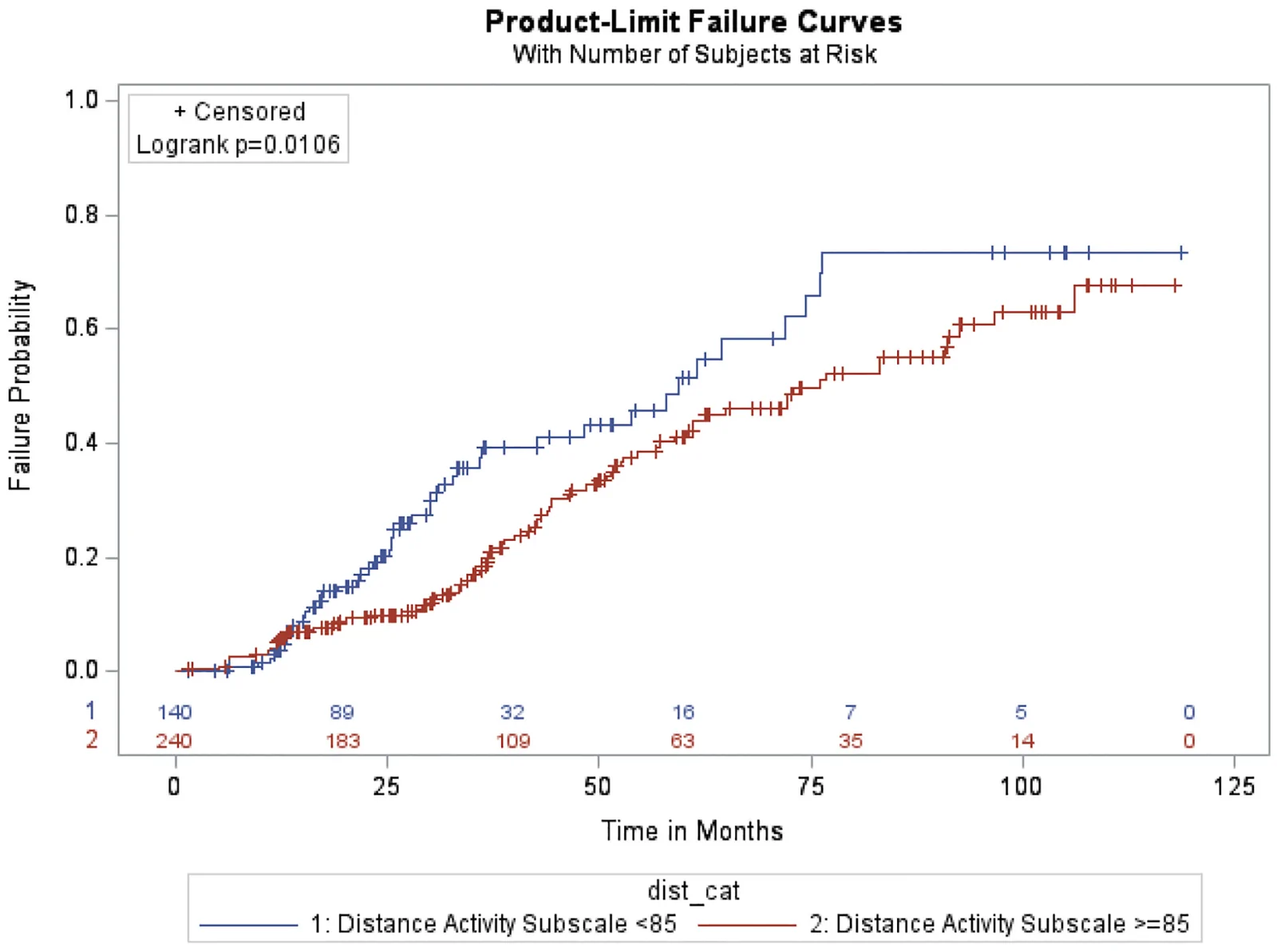
Kaplan-Meier curve of progression to advanced AMD by subscale score of distance activities.

Subscale scores were 85 or higher among 43 (11.3%) patients for general vision and 240 (63.2%) patients for distance vision. Figures 1 and 2 present Kaplan-Meier curves which illustrate time to progression for categorized general vision and distance activities, respectively. Progression rates were significantly higher for patients with scores under 85, for both general vision (*p* = 0.013) and distance activities (*p* = 0.011).

## Discussion

This study investigated the predictive utility of the National Eye Institute Visual Function Questionnaire (VFQ-25) in identifying individuals at risk of progressing to advanced subtypes of age-related macular degeneration (AMD). Among the subscales, distance activities and general vision were most strongly associated with disease progression. However, after adjusting for patient age and LogMAR visual acuity in both eyes, only the general vision subscale remained a statistically significant predictor of progression. Interestingly, visual acuity was no longer significant in the multivariable model. Given that difficulties with distance activities can often be mitigated by compensatory strategies such as moving closer to an object, patients may not perceive deficits in distance vision until more advanced stages of disease progression, which may explain the loss of significance after adjusting for visual acuity.

These findings suggest that self-reported general vision may serve as a meaningful, low-cost indicator of elevated AMD progression risk The general vision subscale is derived from a single question: “At the present time, would you say your eyesight using both eyes (with glasses or contact lenses, if you wear them) is excellent, good, fair, poor, or very poor, or are you completely blind?”. Notably, this one question was found to be a significant predictor of progression, highlighting the potential value of incorporating patient-reported perception of vision into progression risk assessment.

Furthermore, visual acuity is typically assessed using Snellen charts, offering a quick and accessible way to evaluate vision. However, this method is relatively crude and may not fully capture the extent of visual dysfunction.^13^ Oxidative damage and chronic inflammation – key drivers in the pathogenesis of AMD – lead to photoreceptor apoptosis and degeneration of the RPE, resulting in progressive visual impairment that may not be reflected in standard acuity tests, even when patients perceive changes in vision.^14^ As a result, clinically significant declines in function may be overlooked when patient-reported symptoms are not considered and visual acuity is used as the sole measure of visual performance. To address these limitations, clinicians often rely on more comprehensive assessments such as reading speed, contrast sensitivity, low luminance visual acuity (LLVA), and microperimetry, which provide a more nuanced evaluation but are resource-intensive.^13,15–17^ In contrast, a single general vision component of the VFQ-25 questionnaire offers a practical and efficient alternative, capturing meaningful functional changes based on patients’ perceptions without the burden of extensive testing.

Current predictive approaches for AMD progression rely heavily on high-resolution imaging. For example, optical coherence tomography (OCT) and color fundus photography (CFP) have been used to identify biomarkers such as reticular pseudodrusen, which are associated with geographic atrophy development.^10^ Other studies have incorporated genetic data and imaging features, including drusen size and quantification, to enhance predictive models.^18^ A previous study from our group by Gnanaraj et al., identified several multimodal imaging that were markers of progression from iAMD to advanced forms of the disease.^19^ While these methods are effective, they often require costly equipment and specialized expertise, which may limit their accessibility, particularly in resource-limited settings or referral centers such as optometry offices. Furthermore, as a patient-reported outcome measure, the VFQ-25 may capture subjective visual impairment not fully reflected by objective clinical metrics. Ultimately, the present study’s findings contribute to a growing body of research exploring more accessible alternatives for predicting progression and highlight the potential of integrating patient-reported vision into early risk stratification models.

While self-reported general vision remained a significant predictor of AMD progression, other subscales such as near vision were not significantly associated with disease advancement, which was an unexpected finding, potentially warranting further research. Additionally, other VFQ-25 domains such as ocular pain and peripheral vision do not directly reflect macular function, which may account for their lack of significance in predicting conversion. Mental health demonstrated a near-significant association with progression, suggesting a link between vision loss and psychosocial burden. One of our prior studies also demonstrated a significant association between the mental health subscale and AMD classification, further reinforcing the relationship between vision loss and psychosocial outcomes.^20^ Given the substantial impact of AMD on visual function, declines in quality of life are common as patients lose the ability to perform essential tasks, such as driving. These findings highlight the importance of considering the broader effects of disease progression on patients’ overall well-being and support the integration of holistic, patient-centered approaches in the management of AMD.

Despite the strengths of its prospective nature and frequent use of multimodal imaging to monitor AMD progression, the study has some limitations. The relatively small sample size of this study may have introduced inherent bias, potentially reducing the internal validity of the findings. Additionally, the small sample size may have contributed to the wide confidence intervals. Furthermore, since participants were drawn from a registry at the University of Colorado, the study population lacks geographic diversity, potentially limiting the generalizability of findings to the greater AMD population.

Although this study has limitations, it introduces a simple and pragmatic approach to AMD risk assessment. Incorporating VFQ-25 general vision scores into routine clinical workflows may facilitate earlier identification of high-risk patients, particularly in settings where access to advanced diagnostic tools is limited. This strategy could support more equitable care, reduce healthcare costs, and expand preventive efforts in aging populations. Ultimately, this study supports the potential of the VFQ-25 general vision subscale as a scalable, patient-centered tool for early risk assessment in AMD, with meaningful implications for improving access and equity in ophthalmologic care. Future research should focus on validating these findings in larger, more diverse cohorts to clarify the generalizability of VFQ-25 subscale performance across populations.

## Figures and Tables

**Table 1.** Progression rate and hazard ratio by demographic clinical factors for iAMD cases.

	Total Cohort # (column %)	Converted # (row %)	Hazard Ratio 95% CI	*p*-value
All Patients	380	131 (34.5%)	–	–
Age at enrollment, mean (SD)	76.1 (7.1)	76.2 (7.2)	1.02 (1.00, 1.05)	0.096
Sex				
Male	134 (35.3%)	53 (39.6%)	Reference	–
Female	246 (64.7%)	78 (31.7%)	0.79 (0.56, 1.12)	0.184
Race/ethnicity				
White	364 (95.8%)	127 (34.9%)	Reference	–
African-American	5 (1.3%)	2 (40.0%)	0.76 (0.28, 2.06)*	0.592*
Hispanic	6 (1.6%)	2 (33.3%)		
Other	5 (1.3%)	0 (0%)		
Marital Status				
Married	248 (65.3%)	93 (37.5%)	Reference	–
Not married	132 (34.7%)	38 (28.8%)	1.14 (0.78, 1.66)	0.510
Smoking				
Never	196 (51.6%)	73 (37.2%)	Reference	–
Former/current	184 (48.4%)	58 (31.5%)	0.86 (0.60, 1.21)	0.374
Family history of AMD				
Yes	155 (40.8%)	56 (36.1%)	1.16 (0.80, 1.68)	0.442
No	167 (44.0%)	56 (33.5%)	Reference	–
Not sure	58 (15.3%)	19 (32.8%)	1.14 (0.68, 1.92)	0.622
History of condition:				
Diabetes	56 (14.7%)	19 (33.9%)	1.04 (0.64, 1.69)	0.871
Treated hypertension	210 (55.3%)	78 (37.1%)	1.22 (0.86, 1.74)	0.261
LogMAR for better-seeing eye				
Mean (SD)	0.11 (0.12)	0.11 (0.11)	6.39 (1.57, 26.0)	0.001
Median (IQR)	0.10 (0, 0.18)	0.10 (0, 0.18)		
LogMAR for worse-seeing eye				
Mean (SD)	0.26 (0.22)	0.25 (0.22)	2.64 (1.26, 5.55)	0.010
Median (IQR)	0.30 (0.10, 0.30)	0.18 (0.10, 0.30)		

* White versus non-White for statistical comparisons.

**Table 2.** Composite and subscale VFQ-25 scores by progression status for AMD patients.

	Converted Mean (SD) Median (IQR)	Did not Convert Mean (SD) Median (IQR)	Hazard Ratio* 95% CI	*p*-value
Composite Score	89 (10)	88 (12)	0.86 (0.74, 1.02)	0.0752
	93 (86, 96)	92 (83, 96)		
Subscales Scores				
General Health	69 (22)	69 (22)	0.98 (0.91, 1.06)	0.622
	75 (50, 75)	75 (50, 75)		
General Vision	76 (13)	77 (14)	0.84 (0.74, 0.95)	0.006
	80 (60, 80)	80 (80, 80)		
Ocular Pain	91 (15)	88 (16)	1.02 (0.90, 1.16)	0.726
	100 (88, 100)	100 (75, 100)		
Near Activities	88 (18)	85 (18)	0.92 (0.83, 1.01)	0.092
	100 (83, 100)	92 (67.5, 100)		
Distance Activities	87 (17)	86 (18)	0.89 (0.80, 0.98)	0.019
	92 (83, 100)	92 (83, 100)		
Social Functioning	97 (10)	97 (10)	0.90 (0.76, 1.08)	0.261
	100 (100, 100)	100 (100, 100)		
Mental Health	85 (17)	83 (20)	0.91 (0.82, 1.01)	0.064
	88 (81, 100)	88 (81, 94)		
Role Limitations	89 (19)	86 (22)	0.97 (0.89, 1.06)	0.531
	100 (88, 100)	100 (75, 100)		
Dependency	96 (9)	95 (15)	0.93 (0.80, 1.08)	0.345
	100 (100, 100)	100 (100, 100)		
Driving	84 (21)	79 (23)	0.99 (0.91, 1.08)	0.856
	92, (75, 100)	83 (66, 100)		
Color Vision	98 (8)	98 (9)	0.90 (0.75, 1.07)	0.224
	100 (100, 100)	100 (100, 100)		
Peripheral Vision	88 (23)	92 (18)	0.98 (0.86, 1.12)	0.767
	100 (75, 100)	100 (75, 100)		

* Hazard ratio per 10 points on the VFQ.

**Table 3.** Adjusted* hazard ratios of progression for composite and subscale VFQ-25 scores that were significant in univariate models.

	Adjusted* Hazard Ratio** (95% CI)	*p*-value
Model with subscale of general vision		
General Vision	0.85 (0.75, 0.98)	0.022
Age	1.02 (0.99, 1.05)	0.166
Better-seeing eye	2.13 (0.38, 12.0)	0.391
Worse-seeing eye	1.62 (0.63, 4.20)	0.320
Model with subscale of distance activities		
Distance Activities	0.91 (0.82, 1.01)	0.071
Age	1.02 (0.99, 1.04)	0.258
Better-seeing eye	2.32 (0.42, 12.8)	0.335
Worse-seeing eye	1.78 (0.70, 4.52)	0.225

* Adjusted for clinically important variables and variables significantly associated with time to progression in univariate models: age, HVA of better-seeing eye, HVA of worse-seeing eye.

** Hazard ratio per 10 points on the VFQ.
